# Enhancement of Amyloid β_1–43_ Production in the Lens Epithelium of Japanese Type 2 Diabetic Patients

**DOI:** 10.3390/biomedicines8040087

**Published:** 2020-04-13

**Authors:** Yu Mano, Hiroko Otake, Teppei Shibata, Eri Kubo, Hiroshi Sasaki, Noriaki Nagai

**Affiliations:** 1 Faculty of Pharmacy, Kindai University, 3-4-1 Kowakae, Higashi-Osaka, Osaka 577-8502, Japan; yu1215mo26@yahoo.co.jp (Y.M.); hotake@phar.kindai.ac.jp (H.O.); 2 Department of Ophthalmology, Kanazawa Medical University, 1-1 Daigaku Uchinada-machi, Kahoku-gun, Ishikawa 920-0293, Japan; prefse74@yahoo.co.jp (T.S.); kuboe@kanazawa-med.ac.jp (E.K.); sasaki-h@k5.dion.ne.jp (H.S.)

**Keywords:** amyloid β-protein, human lens epithelium, diabetic cataract, type 2 diabetes mellitus, Japanese

## Abstract

We investigated whether the accumulation of amyloid β-protein (Aβ) is enhanced in the lenses of diabetic patients. Lens epithelium samples were collected from Japanese patients during cataract surgery, and the Aβ levels and gene expression of Aβ-producing and -degrading enzymes in the samples were measured by ELISA and real-time RT-PCR, respectively. The Aβ_1–43_ levels in lenses of non-diabetic patients were low (0.11 pmol/g protein), while the levels in lenses of diabetic patients were significantly (6-fold) higher. Moreover, the Aβ_1–43_/total-Aβ ratio in the lenses of diabetic patients was also significantly higher than non-diabetic patients (*p* < 0.05). In addition, the mRNA levels for Aβ-producing enzymes were also enhanced in the lenses of diabetic patients. In contrast to the results for Aβ-producing enzymes, the mRNAs for the Aβ-degrading enzymes in the lenses of diabetic patients were significantly lower than in non-diabetic patients (*p* < 0.05). Furthermore, Aβ_1–43_/total-Aβ ratio in lenses was found to increase with plasma glucose level. In conclusion, these results suggest that high glucose levels cause both an increase in Aβ production and a decrease in Aβ degradation, and these changes lead to the enhancement in Aβ_1–43_ accumulation in the lenses of diabetic patients. These findings are useful for developing therapies for diabetic cataracts and for developing anti-cataract drugs.

## 1. Introduction

The production of amyloid β-protein (Aβ) results from the amyloidogenic processing of amyloid precursor protein (APP), which is cleaved by β-secretase (β-site APP-cleaving enzyme-1, BACE1) and γ-secretase (presenilin-1; PS1) [1,2]. BACE1 cleaves APP at its β-site (the N-terminus of the Aβ domain), the first and critical step in Aβ production [3]. α-Secretase (disintegrin and metalloprotease domain protease 10, ADAM10) is also related to Aβ production. α-Secretase cleaves the α-site within the Aβ domain to produce a soluble secretory APP (sAPPα) [4,5], which reduces Aβ production. Aβ fragments are mainly 39–43 residues in length, and Aβ fragments comprising amino acids 1 to 40, 42, 43 (Aβ_1–40_, Aβ_1–42_, Aβ_1–43_) show cell toxicity via aggregation. Aβ_1–42_ is more hydrophobic and oligomerizes more rapidly in comparison with Aβ_1–40_, and as a result, shows higher cell toxicity [6,7]. Moreover, Aβ_1–43_, compared to Aβ_1–42_, has an additional threonine at the C-terminal produced through an alternative γ-secretase cleavage of APP, and is generally considered to be more aggregation-prone and toxic than Aβ_1–42_ [8,9,10]. In general, it is known that an enhancement in Aβ_1–42_ and Aβ_1–43_ production is related to Alzheimer’s disease. The expressions of β-secretase and γ-secretase in mammalian lenses have been observed [11,12], and the accumulation of Aβ_1–40_ and Aβ_1–42_ has been reported in the cytosol of lens fiber cells from people with Alzheimer’s disease [13]. We also reported that Aβ accumulates in the lens epithelium of humans with cortical opacification (senile cataracts) [14], and showed that the accumulation of Aβ in the lens accelerates lens opacification via nitric oxide-Aβ positive feedback [15]. Thus, these previous reports suggest that the accumulation of Aβ will be related to the onset of senile cataracts in studies using human lens samples. In addition, we found that high glucose levels in the aqueous humor enhance Aβ production, and that elevated Aβ levels in lenses correlate with accelerated lens opacification in diabetic model rats, Otsuka Long-Evans Tokushima Fatty (OLETF) rats, a model of human type 2 diabetes mellitus (DM) [16]. In the relationships between Aβ production and diabetic hyperglycemia, other researchers have also found that a decrease in insulin-degrading enzyme (IDE) activity under high glucose conditions reduces the degradation and elimination of Aβ from the brain [17,18]. Further, it has been reported that the risk of Alzheimer’s disease via Aβ accumulation is increased by 60% in patients with type 2 DM (or insulin-independent DM) over non-diabetics [19]. However, a recent study did not find an association between Aβ levels and hyperglycemia in human lenses. Therefore, it is important to investigate the changes in Aβ production and accumulation in the lenses of diabetic patients. In this study, we investigated whether the accumulation and ratio of Aβ_1–40_, Aβ_1–42_, and Aβ_1–43_ is enhanced in human lenses, and measured the changes in Aβ-producing and -degrading enzymes in the lenses of diabetic patients.

## 2. Materials and Methods

### 2.1. Reagents

RNA later^®^ Solution, the RNeasy Min Kit, and the RNase-Free DNase Set were purchased from Qiagen (Tokyo, Japan). The RNA PCR kit was provided by Takara Bio Inc. (Shiga, Japan). LightCycler FastStart DNA Master SYBR Green I was obtained from Roche Diagnostics Applied Science (Mannheim, Germany). The Human β Amyloid (40) ELISA Kit (dynamic range 1–100 pM) and Human β Amyloid (42) ELISA Kit (dynamic range 0.1–20 pM) were purchased from Wako Pure Chemical Industries (Osaka, Japan), and the Human Amyloid β (1–43) (FL) Assay kit (dynamic range 0.51–32.5 pM) was purchased from IBL (Gunma, Japan). All other chemicals used were of the highest purity commercially available.

### 2.2. Collection of Lens Epithelium Samples

The enucleated lens epithelium samples were obtained from non-Alzheimer’s patients at Kanazawa Medical University (Ishikawa, Japan); Table 1 shows the donor (patient) characteristics. Lens epithelium from normal patients was collected during cataract surgery combined with vitrectomy for macular hole or epiretinal membrane, and used as the non-cataractous control (clear, transparent lenses). Opaque lens epithelium from Japanese patients with (opaque + DM) or without (opaque) type 2 DM was obtained from patients undergoing cataract surgery. The samples were stored in liquid nitrogen or RNA later^®^ Solution. Aβ protein levels and gene expression was measured by ELISA and real-time PCR methods, respectively. In this study, the enucleated lens epithelium with or without nuclear opacification (NUC), posterior subcapsular opacification (PSC), retrodots (RD) and/or water clefts (WC) (mixed-cataracts without cortical opacification) were screened for visual acuity in the clinic prior to surgery, and collected. The cataract types were determined according to the WHO classification and Kanazawa Medical University classification criteria [20]. All experiments were performed in accordance with the Kanazawa Medical University Research Ethics Committee (project identification code 96, 28 May 2014), and Kindai University School of Pharmacy Committee for Research Ethics (project identification code 13–046, 7 September 2013).

### 2.3. Measurement of Aβ-Producing and -Degrading Enzyme mRNAs by a Quantitative Real-Time RT-PCR Method

Gene expression was measured using LightCycler DX 400 according to the manufacturer’s instructions and our previous reports [15]. Briefly, total RNA was extracted from enucleated lens epithelium samples, and purified using an RNeasy Min Kit and RNase-Free DNase Set. The RT and PCR reactions were performed using an RNA PCR kit and LightCycler FastStart DNA Master SYBR Green I, respectively. The RT reaction was performed at 42 °C for 15 min, followed by 5 min at 95 °C. The PCR conditions were as follows: 95 °C for 10 min (Hot start), 60 cycles of 95 °C for 10 s (denaturing), 63 °C for 10 s (annealing), and 72 °C for 5 s (extension). Table 2 shows the sequences of the specific primers (final concentration 10 pmol) used for real-time RT-PCR analysis. The differences in the threshold cycles for target groups [APP, BACE1, PS1, ADAM10, neprilysin (NEP), and endothelin converting enzyme-1 (ECE-1)] and β-actin were used to calculate the levels of mRNA expression in human lens epithelium samples.

### 2.4. Measurement of Aβ_1–40_, Aβ_1–42_, and Aβ_1–43_ by ELISA Methods

Aβ_1–40_ Aβ_1–42_ and Aβ_1–43_ levels were measured using a Human β Amyloid (40) ELISA Kit, Human β Amyloid (42) ELISA Kit, and Human Amyloid β (1–43) (FL) Assay kit, respectively, according to the manufacturers’ instructions and our previous reports [15]. Briefly, the enucleated lens epithelium samples were homogenized in 70% formic acid (250 µL), and centrifuged at 9100× *g* for 15 min at 4 °C. The supernatants were diluted into 1 M Tris base (4750 µL), and the mixtures were used for the measurements of Aβ_1–40,_ Aβ_1–42_, and Aβ_1–43_. Aβ levels are expressed as pmol/g protein, and total-Aβ was estimated as the sum of Aβ_1–40_, Aβ_1–42_ and Aβ_1–43_ (Aβ_1–40_ + Aβ_1–42_ + Aβ_1–43_). In addition, the ratios of Aβ_1–40_, Aβ_1–42_, and Aβ_1–43_ accumulation were analyzed as Aβ_1–40_/total-Aβ, Aβ_1–42_/total-Aβ, and Aβ_1–43_/total-Aβ, respectively.

### 2.5. Statistical Analysis

All data are expressed as the mean ± S.D. Unpaired Student’s, Aspin-Welch’s *t*-test or Dunnett’s multiple comparison was used to evaluate statistical comparisons. *p* values less than 0.05 were considered statistically significant.

## 3. Results

### 3.1. Accumulation of Aβ in the Lens Epithelium of Diabetic Patients

**Figure 1:**
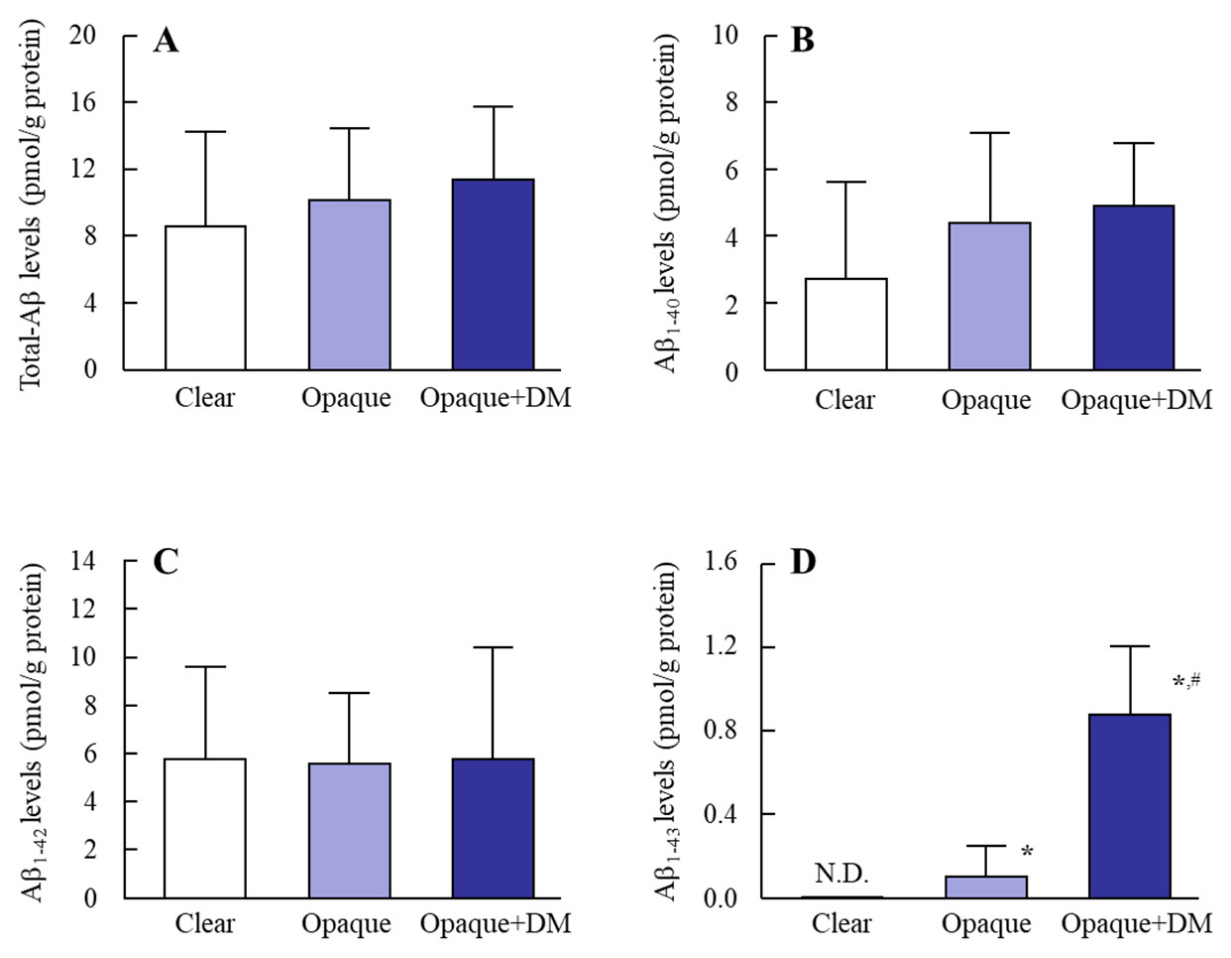
Changes in total-Aβ (**A**), Aβ_1–40_ (**B**), Aβ_1–42_ (**C**), and Aβ_1–43_ (**D**) in lens epithelium of diabetic patients. Patient characteristics are shown in Table 1 (ELISA method group). Open columns, transparent lens epithelium; closed columns, opaque lens epithelium. Clear, transparent lens epithelium; opaque, opaque lens epithelium; opaque + DM, opaque lens epithelium with type 2 DM. *n*.D., not detectable. *n* = 5–18. * *p* < 0.05, vs. clear for each group. # *p* < 0.05, vs. opaque for each group.

**Figure 2:**
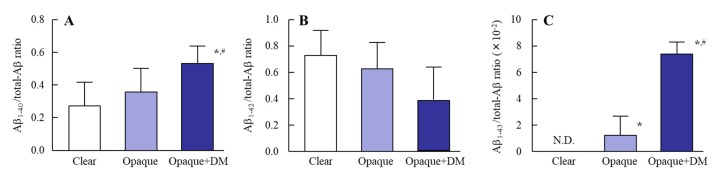
Changes in Aβ_1–40_/total-Aβ (**A**), Aβ_1–42_/total-Aβ (**B**), and Aβ_1–43_/total-Aβ (**C**) ratios in lens epithelium of diabetic patients. Patient characteristics are shown in Table 1 (ELISA method group). Open columns, transparent lens epithelium; closed columns, opaque lens epithelium. Clear, transparent lens epithelium; opaque, opaque lens epithelium; opaque + DM, opaque lens epithelium with type 2 DM. N.D., not detectable. *n* = 5–18. * *p* < 0.05, vs. clear for each group. # *p* < 0.05, vs. opaque for each group.

Figure 1 and Figure 2 show the Aβ levels (Figure 1) and Aβ/total-Aβ ratios (Figure 2) in the lens epithelium of patients with or without type 2 DM. The total-Aβ levels were similar among transparent lenses and opaque lenses with or without DM. On the other hand, the ratio of Aβ_1–40_ accumulation (Aβ_1–40_/total-Aβ) were higher in the lenses of diabetic patients. No Aβ_1–43_ in transparent lenses was detected, while the Aβ_1–43_ levels in opaque lenses from non-diabetic patients was low (0.11 ± 0.12 pmol/g protein). In contrast to the results in transparent lenses and opaque lenses without DM, the Aβ_1–43_ levels in opaque lenses from diabetic patients was significantly increased, and the Aβ_1–43_ levels in opaque DM lenses was 6.0-fold higher than in opaque lenses from non-diabetic patients.

### 3.2. Changes in Levels of Aβ-Producing and –Degrading Enzyme mRNAs in the Lens Epithelium of Diabetic Patients

**Figure 3:**
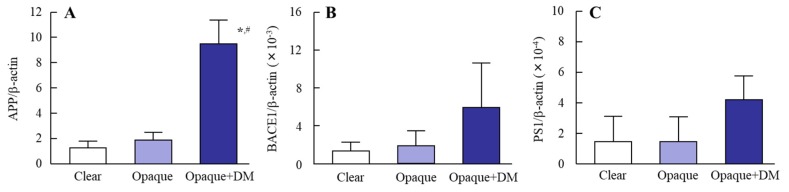
Changes in APP (**A**), BACE1 (**B**), and PS1 (**C**) mRNAs in lens epithelium of diabetic patients. Patient characteristics are shown in Table 1 (PCR method group). Open columns, transparent lens epithelium; closed columns, opaque lens epithelium. Clear, transparent lens epithelium; opaque, opaque lens epithelium; opaque + DM, opaque lens epithelium with type 2 DM. *n* = 3–15. * *p* < 0.05, vs. clear for each group. # *p* < 0.05, vs. opaque for each group.

**Figure 4:**
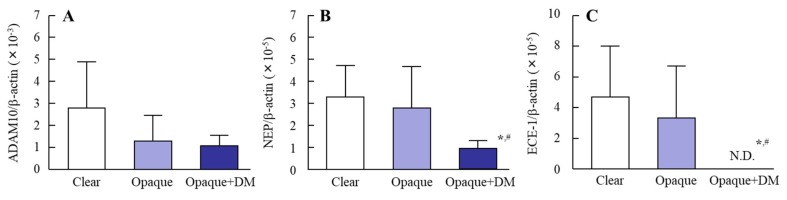
Changes in ADAM10 (**A**), NEP (**B**), and ECE-1 (**C**) mRNAs in lens epithelium of diabetic patients. Patient characteristics are shown in Table 1 (PCR method group). Open columns, transparent lens epithelium; closed columns, opaque lens epithelium. Clear, transparent lens epithelium; opaque, opaque lens epithelium; opaque + DM, opaque lens epithelium with type 2 DM. N.D., not detectable. *n* = 3–15. * *p* < 0.05, vs. clear for each group. # *p* < 0.05, vs. opaque for each group.

Figure 3 shows the levels of Aβ-producing enzyme mRNAs (APP, BACE1, and PS1) in the lens epithelium of patients with or without type 2 DM. The expressions of APP, BACE1 and PS1 showed no significant differences between transparent lenses and opaque lenses from non-diabetic patients. On the other hand, the levels of Aβ-producing enzyme mRNAs were increased in the lens epithelium of diabetic patients, and the level of APP mRNA in opaque lenses with DM was significantly higher than in transparent and opaque lenses without DM. Figure 4 shows the levels of Aβ-degrading enzyme mRNAs (ADAM10, NEP, and ECE-1) in the lens epithelium of patients with or without type 2 DM. Although the ADAM10 mRNA levels in opaque lenses with or without DM tended to be lower than that in transparent lenses, the difference was not significant. The NEP and ECE-1 mRNA levels in opaque lenses were similar to those in transparent lenses from non-diabetic patients. On the other hand, the NEP mRNA level in opaque lenses from diabetic patients was significantly lower than in transparent or opaque lenses from non-diabetic patients. In addition, the ECE-1 mRNA was not detected in opaque lenses from diabetic patients. Table 3 shows the relationship between plasma glucose and Aβ production and degradation in patients with or without cataracts and DM. A significant difference was observed in Aβ_1–43_ accumulation vs. glucose level. In addition, the levels of Aβ-producing mRNA (APP and BACE1) also increased with the plasma glucose levels. In contrast to the results for Aβ_1–43_ accumulation and Aβ-producing enzyme mRNA, there were no significant differences between the levels of Aβ-degrading enzyme mRNAs (ADAM10, NEP, and ECE-1) and plasma glucose level. In this study, we measured the correlation between Aβ_1–43_ and plasma glucose in the lens epithelium of diabetic patients, and found a significant increase in the Aβ_1–43_ ratio (Aβ_1–43_/total-Aβ level) with plasma glucose level (= 0.0033*x* + 0.0216, *r* = 0.8359).

## 4. Discussion

**Scheme 1:**
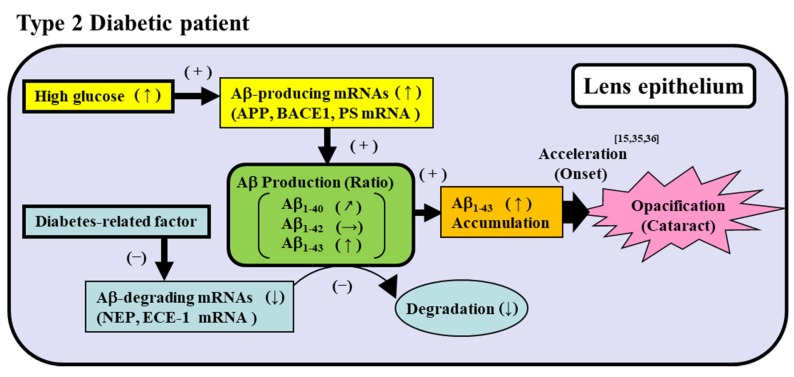
Relationship between Aβ_1–43_ accumulation and lens opacification in diabetic patients.

Since humans have a long lifespan [21], the proteins in the lens eventually precipitate to form aggregates due to ultraviolet light exposure resulting in lens opacification that causes blurred vision and blindness [22], and this is exasperated in patients with diabetes. As one factor in cataract development, we previously reported that Aβ accumulation in the lens accelerates lens opacification via nitric oxide-Aβ positive feedback [15]. In addition, we found that high glucose levels in the aqueous humor enhance Aβ production, and elevated Aβ levels in the lens cortex correlate with accelerating opacification in OLETF rats, a model of human type 2 DM [16]. However, there are no reports concerning Aβ levels in the lenses of patients with DM. In the present study, we investigated the changes in Aβ accumulation in the lenses of diabetic patients, and found that an increase in Aβ-producing enzyme mRNAs and a reduction in Aβ-degrading enzyme mRNAs as compared to non-diabetic patients, resulting in an enhancement in Aβ_1–43_ production in the lens epithelium. These elevated Aβ_1–43_ levels may be related to the acceleration of lens opacification in diabetic patients (Scheme 1).

First, we investigated whether Aβ_1–40_, Aβ_1–42_, and Aβ_1–43_ accumulate in the lens epithelium of diabetic patients. In a previous study to measure Aβ production and accumulation in human lenses, we showed that the production and accumulation of Aβ are similar between normal patients (transparent lenses) and opaque lenses from cataract patients with NUC, PSC, RD and/or WC. However, Aβ accumulation was enhanced in the lens epithelium of humans with cortical cataracts [14]. Based on these findings, we used the lens epithelium from patients with NUC, PSC, RD and/or WC as opaque lenses (mixed-cataracts without cortical opacification). The ratios of Aβ_1–40_ and Aβ_1–43_ accumulation were enhanced in the lenses from diabetic patients (Figure 1 and Figure 2). We previously reported that the levels of Aβ-producing enzyme mRNAs (APP, BACE1, and PS1) are enhanced under high glucose conditions, resulting in Aβ accumulation in the lens cortex of OLETF rats and cultured human lens epithelial cells [16]. Therefore, we attempted to measure the changes in Aβ-producing enzyme mRNAs in the lenses of diabetic patients, and found enhancements in APP, BACE1, and PS1 in the lens epithelium of diabetic patients (Figure 3). It has been reported that the ratios of Aβ_1–42_ and Aβ_1–43_ production are enhanced by abnormal changes in APP and PS1 [23,24,25]. Therefore, the increases in APP and PS1 mRNA may cause the enhancement in the ratio of Aβ_1–43_ production. In addition, both Aβ_1–43_ accumulation and Aβ-producing enzyme mRNA levels increased in correlation with increasing plasma glucose levels in human lens epithelium (Table 3). These results support our previous study using diabetic model rats and human cultured cells [16], and suggest that the high glucose levels increase Aβ production and accumulation via enhanced APP and Aβ-producing enzymes in the lenses of human patients.

Aβ accumulation implies a dynamic imbalance between biosynthesis and removal. Therefore, the measurement of Aβ-degrading enzymes is also important. NEP is a 90–110 kDa plasma membrane glycoprotein member of the neutral zinc metalloendopeptidase family [26,27,28] and has been shown to play a major role in the clearance of Aβ in brain [29]. ECE-1 is type II integral membrane zinc metalloendopeptidase, and also degrades the Aβ peptide [30]. No significant relationship was observed between hyperglycemia and NEP or ECE-1 mRNA levels (Table 3). However, the NEP mRNA level in the lenses of diabetic patients was only 33.8% the level in lens epithelium from non-diabetic patients (opaque group), and no ECE-1 mRNA was detected diabetic lenses by 60 cycles of PCR (Figure 4). It has been reported that the inhibition of NEP improves whole body insulin-mediated glucose disposal, and that the inhibition of both angiotensin-converting enzyme and NEP in obese insulin-resistant Zucker rats induces insulin sensitization and increased myocardial glucose uptake [31,32,33]. These reports suggest that NEP contributes directly to the development of insulin resistance, and that negative-feedback may be related to the reduction in NEP mRNA levels in the lens epithelium of diabetic patients in this study. However, this remains a hypothesis, and the mechanism for the reduction in Aβ-degrading enzymes requires further examination. On the other hand, in a study using an animal diabetic model (streptozotocin-induced diabetic rats, STZ rats), Liu et al. [34] showed that NEP and ECE-1 mRNA levels were significantly decreased in the hippocampus, and slightly decreased in brain cortex. Moreover, ECE-1 activity was significantly decreased in both the hippocampus and cortex of STZ rats, while NEP activity was slightly decreased in both brain regions [34]. These results in STZ rat brain are similar to our results in lens epithelium of diabetic patients. Therefore, it is possible that the mechanism for the reduction in NEP and ECE-1 mRNAs can be elucidated by further studies using STZ rats.

Normal eye lenses are transparent and focus light onto the retina, and high protein concentrations within the lens capsule preserve the refractive index, which maintains focus [35]. However, it is known that the accumulation of Aβ induces excessive reactive oxygen species, such as nitric oxide, and causes lens opacification [15,36,37]. In addition, Xu et al. reported that Aβ expression levels are enhanced in patients with age-related cataracts, and Aβ production in the early and mid-stages of age-related cataract development is one of the potential mechanisms for the accelerating oxidative stress during cataractogenesis [38]. Thus, Aβ contributes to the onset of cataracts. Taken together, we hypothesize that an increase in Aβ-producing enzymes and a decrease in Aβ-degrading enzymes take place in the human lens epithelium of diabetic patients. The imbalance between biosynthesis and removal causes the changes in the levels and ratio of Aβ_1–40_, Aβ_1–42_, and Aβ_1–43_, resulting in an enhancement in Aβ_1–43_ production. The elevated Aβ_1–43_ levels may accelerate the onset of cataracts (lens opacification) in diabetic patients (Scheme 1). On the other hand, it is important to investigate Aβ accumulation in the lenses of diabetic patients with cortical opacification, since the above findings were obtained from mixed-cataracts without cortical opacification. We measured Aβ_1–43_ levels in the lens epithelium of diabetic patients with cortical opacification in this study (cortical opacification 2.0 ± 0.7, NUC 0.8 ± 0.8, PSC 0.5 ± 1.0, RD 1.0 ± 1.2, WC 1.3 ± 1.2, age 74.8 ± 8.6, *n* = 6, Male 1, Female 5), and the Aβ_1–43_ levels (Aβ_1–43_, 2.9 ± 1.1 pmol/g protein, Aβ_1–43_/total-Aβ 21.5 ± 7.1 × 10^−2^) were higher than in mixed-cataracts without cortical opacification (Aβ_1–43_, 1.83 ± 1.8 pmol/g protein, Aβ_1–43_/total-Aβ 8.8 ± 1.2 × 10^−2^) [14]. These results show that hyperglycemia may also accelerate cortical opacification in diabetic patients.

Further studies are needed to clarify the precise mechanism for the enhanced Aβ_1–43_ level and ratio, and the reduced Aβ-degrading enzyme level in lens epithelium of diabetic patients. In addition, it is known that IDE and matrix metalloproteinase 9 (MMP-9) are also involved in the degradation of Aβ [39]. Therefore, we are now investigating changes in IDE and MMP-9 in the lens epithelium of diabetic patients, and will attempt to demonstrate the relationship between diabetes-related factors and Aβ production and accumulation in the lenses of STZ rats.

## 5. Conclusions

We measured Aβ_1–40_, Aβ_1–42_, and Aβ_1–43_ accumulation in the lens epithelium of Japanese type 2 diabetic patients, and found that Aβ_1–43_ production and accumulation in diabetic patients are higher than in non-diabetic patients. In addition, we show that the enhanced Aβ_1–43_ ratio may be related to the onset and/or acceleration of diabetic cataracts. These findings support our previous studies [14,15,16], and may be useful in developing therapies for diabetic cataracts.

## Figures and Tables

**Table 1:** The characteristics of the donors in this study.

Characteristic	ELISA Method			PCR Method		
	Clear	Opaque	Opaque + DM	Clear	Opaque	Opaque + DM
Age (y)	62.2 ± 14.9	73.9 ± 9.9	74.2 ± 7.8	64.1 ± 15.9	68.8 ± 13.8	69.3 ± 6.6
Glucose (mg/dL)	107.5 ± 16.3	102.3 ± 18.6	174.6 ± 72.6	97.7 ± 19.9	111.9 ± 41.9	247.3 ± 42.0
Total opacification	0	3.2 ± 1.6	3.2 ± 0.9	0	3.2 ± 1.5	6.0 ± 1.3
Cortical opacification	0	0	0	0	0	0
Nuclear opacification	0	1.0 ± 0.7	0.2 ± 0.3	0	0.8 ± 0.6	0.7 ± 0.3
Posterior subcapsular opacification	0	0.5 ± 1.0	0.6 ± 0.5	0	1.0 ± 1.0	0.3 ± 0.4
Retrodots	0	1.2 ± 1.4	1.8 ± 0.7	0	0.5 ± 0.6	3.0 ± 0.0
Water clefts	0	0.4 ± 0.6	0.6 ± 0.9	0	0.9 ± 1.0	2.0 ± 1.3
*n* (60 samples)	Male 6, Female 7	Male 7, Female 11	Male 3, Female 2	Male 3, Female 3	Male 8, Female 7	Male 2, Female 1

Cataract types were determined according to the WHO classification and Kanazawa Medical University classification criteria [20]. Clear, transparent lens epithelium; opaque, opaque lens epithelium; opaque + DM, opaque lens epithelium with type 2 diabetes mellitus.

**Table 2:** Primers used for real-time PCR analysis.

Primer		Sequence (5′-3′)
*APP*	FOR	TGGTGGGCGGTGTTGTCATA
	REV	TGGATTTTCGTAGCCGTTCTGC
*BACE1*	FOR	GCAAGGAGTACAACTATGAC
	REV	AGCTTCAAACACTTTCTTGG
*PS1*	FOR	ATCATCTGCATAGTCCTCTC
	REV	AGACAGCTTTGATGTTCAAG
*ADAM10*	FOR	CACATGATTCTGGAACAGAG
	REV	GTTGTTAAGTTTGTCCCCAG
*NEP*	FOR	CTGATATCAACACTCCAAAGC
	REV	TCATCGTAGGTTGCATAGAG
*ECE-1*	FOR	AGAATGAGATTGTGTTTCCG
	REV	CTATGCCACCAAAGTTTAAGG
*β-actin*	FOR	GTGGCATCCACGAAACTACC
	REV	CAGGGCAGTGATCTCCTTCT

**Table 3:** Approximation formulas and correlation coefficients (*r*) between of plasma glucose and Aβ accumulation in human lens epithelium.

vs. Glucose	Approximation Formula	*r*
Total-Aβ levels	y = −1.3 × 10^−2^x + 11.5	0.089
Aβ_1–40_ levels	y = 4.5 × 10^−3^x + 3.5	0.051
Aβ_1–42_ levels	y = −2.2 × 10^−2^x + 8.4	0.225
Aβ_1–43_ levels	y = 5.3 × 10^−3^x – 0.4	0.579 *
APP mRNA	y = 3.6 × 10^−2^x – 1.8	0.798 *
BACE1 mRNA	y = 0.2 × 10^−5^x – 0.2 × 10^−3^	0.419 *
PS1 mRNA	y = 0.2 × 10^−5^x – 0.1 × 10^−4^	0.320
ADAM10 mRNA	y = −0.2 × 10^−5^x + 0.2 × 10^−3^	0.049
NEP mRNA	y = −0.2 × 10^−7^x + 0.3 × 10^−4^	0.046
ECE1 mRNA	y = −0.5 × 10^−5^x + 0.5 × 10^−4^	0.242

Patient characteristics are shown in Table 1. *n* = 24 (PCR) and 36 (ELISA). * *p* < 0.05.

## Abbreviations

ADAM10	a disintegrin and metalloprotease domain protease 10
APP	amyloid precursor protein
Aβ	amyloid β-protein
BACE1	β site APP cleaving enzyme
DM	diabetes mellitus
ECE	endothelin converting enzyme
IDE	insulin-degrading enzyme
MMP-9	matrix metalloproteinase 9
NEP	neprilysin
NUC	nuclear opacification
OLETF rat	Otsuka Long-Evans Tokushima Fatty rat
PS	presenilin
PSC	posterior subcapsular opacification
RD	retrodots
sAPPα	soluble secretory APP
STZ rat	streptozotocin-induced diabetic rat
WC	water clefts
r	correlation coefficient
